# Immediate Beverage Reformulation and Sustained Consumer Behavior Change under Chile’s Food Labeling Policies

**DOI:** 10.21203/rs.3.rs-11297879/v1

**Published:** 2026-10-26

**Authors:** Mrignyani Sehgal, Shu Wen Ng, Lindsey Smith Taillie, Guillermo Paraje

**Affiliations:** University of North Carolina at Chapel Hill; University of North Carolina at Chapel Hill; University of North Carolina at Chapel Hill; Universidad Adolfo Ibáñez

**Keywords:** Front-of-package nutrition labeling, Product reformulation, Household purchase behavior, Sugar-sweetened beverages, public health

## Abstract

**Background:**

Chile’s front-of-package warning label law, implemented in phases beginning in 2016, aimed to reduce consumption of foods and beverages high in sugar, sodium, saturated fat, and calories. While previous studies have documented substantial reductions in sugar purchases following the policy, there is limited understanding of the mechanisms, particularly the relative contributions of producer and consumer responses.

**Method:**

This study decomposes changes in the sugar content of beverage purchases into changes made by producers, such as reformulating, introducing, and discontinuing products, and changes in consumer purchasing, such as switching between products and changing the amount purchased. We use household purchase data from Kantar WorldPanel Chile from January 2015 to June 2019, merged with time-specific nutrition facts panel data, to conduct decomposition analyses of approximately 3500 beverage items across three policy periods: pre-policy, Phase 1 (July 2016-June 2018), and Phase 2 (July 2018-June 2019).

**Results:**

Sugar from beverage purchases fell from 17.1 g/capita/day before the policy to 13.2 g/capita/day in Phase 1 and to 11.2 g/capita/day in Phase 2 (totaling 5.9 g/capita/day or 35% of the pre-policy level). In Phase 1, producer and consumer responses contributed roughly equally. Of the 3.9 g/capita/day fall in Phase 1, reduced purchase volumes accounted for 1.2 g and reformulation of continuing products for 1.1 g. Of the additional 2.0 g/capita/day fall in Phase 2, consumer responses dominated, with product switching accounting for 1.1 g/capita/day and reduced volumes for 0.8 g/capita/day, while reformulation contributed only 0.3 g/capita/day. Reductions were driven almost entirely by high-sugar beverages, with minimal reformulation among low-sugar beverages. Patterns were similar across socioeconomic groups, though the highest socioeconomic group saw a larger reduction in sugar purchases.

**Conclusion:**

These findings demonstrate that nutrition labeling policies trigger both immediate producer reformulation and sustained consumer behavior change, highlighting the importance of considering both supply- and demand-side mechanisms in food policy design.

## Background

Globally, excessive sugar intake is a key risk factor for overweight and obesity as well as a range of non-communicable diseases including diabetes and cardiovascular diseases [1]. Chile has the highest obesity prevalence in Latin America [2, 3] and higher body mass index accounted for an estimated 31.6% of deaths from major non-communicable diseases (NCDs) and 20.4% of all deaths in the country in 2018. This burden rose steadily between 2003 and 2017 when the prevalence of obesity among adults increased from 23.2% to 31.2%, while overweight rates climbed from 37.8% to over 39% [4].

One important contributor to this growing burden is the high consumption of sugar-sweetened beverages (SSBs) in Chile, with food expenditure on SSBs growing substantially between 1990 and 2010 [5]. By 2009–2014, Chile had the world’s highest per capita SSB sales and the largest absolute growth in SSB sales of any country [6]. To combat this, Chile passed Ley 20.606 in 2012, alongside a tax on SSBs introduced in 2014, requiring that packaged foods and beverages containing added sugar, added sodium, or added saturated fat and exceeding thresholds for these nutrients or overall calories to carry front-of-package warning labels. The labels consist of black octagon(s) with the text that it is high in sugar, sodium, saturated fat, and/or calories. This was accompanied by restrictions on marketing targeted at children, as well as a ban on the sale in schools of any foods or beverages exceeding specified thresholds for added sugars, sodium, or saturated fats. The policy was implemented in three phases with increasingly strict nutrient thresholds implemented in July 2016 (Phase 1); July 2018 (Phase 2); and July 2019 (Phase 3).

The law has since been extensively evaluated, with studies documenting changes in food and beverage purchases, product reformulation, marketing practices, prices, and, more recently, health outcomes [7-11]. On the consumer side, evaluations have found substantial reductions in purchases of products subject to the policy. Taillie et al. [8] found a 27.5% reduction in calories purchased from high-in beverages following Phase 1, with a smaller increase in purchases of beverages not subject to the policy. Taillie et al. [9] found that three years after implementation, purchases of sugar from high-in foods and beverages declined by 36.8%, equivalent to 30.4 fewer calories per capita per day, alongside reductions in energy, sodium, and saturated fat. Reductions in sugar and energy were primarily driven by beverages, while reductions in sodium and saturated fat were driven by foods.

Evidence on producer responses are more limited [12, 13], even though manufacturers can respond to labelling policy in several ways by adjusting prices, reformulating products, changing which products they sell, altering marketing, and changing package sizes [14]. Reyes et al. [7] found that the share of products carrying at least one “high in” warning label fell from 51% to 44% after Phase 1, with the largest reduction observed for products high in sugar, which declined from 80% to 60%. A longitudinal study comparing products in 2013 and 2019 similarly found a 15% reduction in total sugar content, with particularly large reductions among dairy products, sugary beverages, and other products affected by the regulation. More recent evidence following full implementation found further reductions in sugar content across several food and beverage categories, although reformulation was concentrated among products closer to the regulatory thresholds [15].

Taken together, existing evaluations document substantial changes in products and consumer purchases following Chile’s labeling policy but rarely disentangle the relative contribution of producer and consumer responses. Manufacturers may respond by reformulating products to reduce sugar content or by changing product availability, while consumers may respond by switching to products with fewer warnings or changing the amount they purchase. Existing evaluations largely capture the combined effect of these responses on sugar purchases. As a result, it remains unclear how much of the reduction in sugar purchases can be attributed to changes in products versus changes in consumer purchasing.

Distinguishing these supply- and demand-side channels determines which policy lever matters. If reductions came mainly from reformulation, the operative instrument is the nutrient threshold, and tightening it yields further gains. If they came mainly from switching, the operative instrument is label salience, which depends on sustained consumer attention and may decay as labels become familiar.

This study’s objective was to decompose changes in the sugar content of Chilean household beverage purchases across the first two phases of the labeling law into producer-driven components (reformulation of continuing products and net product entry) and consumer-driven components (switching between products and changes in purchase volumes). A second objective was to assess whether these components differed between higher- and lower-socioeconomic-status households, and between beverages above and below the Phase 2 sugar threshold.

## Methods

### Data

This study uses data on household beverage purchases from January 2015 to June 2019, from the Kantar WorldPanel Chile. We define January 1, 2015, through June 30, 2016, as the pre-policy period because it follows the 2014 sugar-sweetened beverage tax but precedes the implementation of front-of-package warning labels. We define the post-policy period based on the implementation of increasingly strict warning label thresholds: Phase 1 from July 1, 2016, to June 30, 2018, and Phase 2 from July 1, 2018, to June 30, 2019. For beverages, the thresholds for calories decreased from 100 to 80 calories/100 mL and for sugar from 6 to 5 g/100 mL between Phase 1 and Phase 2.

The dataset includes purchases from a panel of over 2,844 Chilean households, designed to represent the urban population. Data were aggregated at the household–month level, resulting in 138,391 observations from cities with populations greater than 20,000 across 13 of Chile’s 16 regions. The dataset covered beverage purchases of 3492 unique beverage items across 100% fruit and vegetable juices, coffee and tea, dairy-based beverages and dairy substitutes, industrialized fruit and vegetable juices, sodas, sports and energy drinks, and bottled water. See Appendix A1 for beverage item identification criteria.

To determine the nutritional content of each household’s beverage purchases over time, we merged purchase data with nutrition facts panel (NFP) data at the product level as described in Kanter et al. [16]. Nutrition Facts Panel (NFP) data were obtained from food package photographs collected by the Institute of Nutrition and Food Technology (INTA) at the University of Chile in supermarkets during the first quarter of each year beginning in 2015, and supplemented with Mintel product data when INTA information was unavailable. Importantly, the linkage was policy-period sensitive. Purchases in each period were matched to NFP data collected during that same period, so that the sugar content assigned to each product reflects the formulation on sale at the time and captures reformulation between periods. See Appendix A2 for details of the linking procedure. This merged dataset provides the sugar concentration of each product, expressed in grams of sugar per 100 ml.

### Analysis

The study looks at product turnover in beverages, specifically how many products continued, were discontinued, or were newly introduced across the pre-policy, Phase 1, and Phase 2 periods. Beverage items were classified as continuing between two periods if they were purchased in both periods, new if they were only purchased in the end period and discontinued if they were only purchased in the base period.

Among continuing beverages, the study examines reformulation at the product level, focusing on whether sugar content decreased, increased, or remained unchanged across the three policy periods. Reformulation was defined as a change in sugar concentration greater than ± 20%, which is in line with current ranges allowed for margins of error in reporting sugar values in the United States. We also rounded sugar concentrations to the nearest 0.1 g/100 ml to mitigate the influence of large percentage changes from low bases on these frequencies.

Importantly, observed reformulation of sugar concentration reflects changes at the product level, not reflective of purchase behavior. It is possible that, although sugar concentration declined primarily in a few products, these were high-selling items that account for large sales volumes due to their frequent purchase. As a result, simply examining the percentage of products that were reformulated does not capture the full picture. To better understand the impact, it is essential to account for purchase volume.

To address this, the study assesses overall reductions in sugar, using the decomposition methodology from Bercholz et al. [17]. It decomposes overall change into components driven by producers (reformulation, product entry and exit), and consumers (changes in purchase volume, and product switching). This approach extends traditional decomposition methods [18-20] by accounting for both nutrient concentration and purchase volume.

Our decomposition unfolds in two stages. First, we separate changes in the sugar concentration of beverages (grams of sugar per 100 ml) into components attributable to consumer behavior and producer reformulation. Because the overall effect of reformulation on sugar purchased depends on the extent to which the reformulated product is purchased, we account for purchase volumes in our second stage of decompositions. This methodology is described in detail in Bercholz et al. [17] and in Appendix A3.

The decomposition distinguishes changes in purchased sugar into producer- and consumer-related components. The producer-side component captures **reformulation**, reflecting changes in sugar concentration among continuing products, and **net product entry**, reflecting the contribution of products entering or exiting the market. The consumer-side component captures **consumer switching and changes in purchase volumes**, reflecting changes in the products purchased and the quantities purchased among continuing products. Finally, each stage of the decomposition generates a **residual cross term.** This term arises because the reformulation and consumer switching effects are calculated while holding either purchase volumes or sugar concentrations constant. The cross term therefore captures the joint effect of changes in sugar concentration and volume shares. A positive cross term indicates that changes in sugar concentration and market share moved in the same direction, such that products that increased in sugar also gained market share, or products that decreased in sugar lost market share. Conversely, a negative cross term indicates that changes moved in opposite directions, such that lower-sugar products gained market share or higher-sugar products lost market share.

Results of this decomposition exercise are expressed as the contribution of each component to the overall change in sugar purchases, reported in g/capita/day and as a percentage of the net change. These percentages indicate the share of the overall reduction attributable to each component. Because each component is estimated independently while holding the other components constant, the reported shares may sum to more than 100%. We report results for each policy phase and cumulatively from pre-policy through Phase 2 to assess producer and consumer responses over time.

We conducted the descriptive and decomposition analyses separately for high- and low-sugar beverages, defined as > or = 5 g and < 5 g of sugar per 100 mL, respectively. The 5 g/100 mL threshold corresponds to the final Phase 2 threshold for a beverage warning label under Chilean regulation. We applied this threshold consistently across all periods for two reasons: first, Phase 2 represents the final determination of which products were classified as unhealthy under the regulation and therefore provides the most relevant benchmark for tracking changes over time; and second, a consistent definition ensures that observed changes in purchases reflect consumer behavior rather than shifts in regulatory thresholds.

### Secondary analysis

The study assesses whether the decomposition results vary by socio-economic groups. Households were categorized by Kantar into 4 SES categories using the 2007 methodology of the market research association Asociacion de Investigadores de Mercado y Opinion Publica de Chile (AIM). SES was measured by categorizing households based on the possession of certain goods (e.g., refrigerator, washing machine, DVD player), payment for specific services (internet and full-time domestic worker), and the education level of the household head, according to Kantar’s methodology. We consolidated the four Kantar SES categories into two groups, combining the two lowest SES segments into a single “low SES” category and the two highest into “high SES”.

## Results

### Descriptive statistics for product turnover

Figure 1 describes frequencies of continuing, discontinued and new beverage items for high sugar and low sugar beverages. High sugar beverages accounted for approximately 20% of products, compared with 80% classified as low sugar beverages. This distribution remained consistent across all phases. We note a decline in newly introduced beverage items as well as significant beverage discontinuation across both policy phases, particularly among high sugar beverages. Specifically, in Phase 1, newly introduced beverages accounted for 22.6% (128 of 565) of high-sugar beverages and 50.5% (1,026 of 2,028) of low-sugar beverages. In Phase 2, these shares declined further, with newly introduced beverages accounting for only 3.9% (13 of 331) of high-sugar beverages and 9.2% (107 of 1,163) of low-sugar beverages. This shift indicates a notable reduction in the introduction of new beverages in the later phase. On the other hand, a significant share of beverage items was discontinued between the pre-policy and Phase 1 periods, with 45.1% of high-sugar beverages (342 of 779) and 45.6% of low-sugar beverages (683 of 1,499) discontinued. A similar pattern was observed from Phase 1 to Phase 2, with 43.7% of high-sugar beverages (247 of 565) and 47.9% of low-sugar beverages (972 of 2,028) discontinued.

### Reformulation among continued products

Figure 2 shows reformulation among number of continued products across policy phases, separately for high sugar and low sugar beverage. In Phase 1, 59% of high sugar beverages and 70% of low sugar beverages were not reformulated, meaning their sugar content remained unchanged. In Phase 2, this share increased even further-80% of high sugar and 87% of low sugar beverages retained their original sugar levels. Among the reformulated items, 14% of high sugar beverages were reformulated to reduce sugar content in Phase 1. Interestingly, 26% high sugar beverages also saw increase in sugar content, in Phase 1 which declined to 15% in Phase 2 and 8% of low sugar beverages increased their sugar content.

### Decomposition of changes in the sugar content of beverage purchases

When accounting for volume of purchases, the decomposition results for all households are shown in Fig. 3. The sugar content of beverage purchases fell by 3.9 g/capita/day between the Pre-policy and Phase 1 period (from 17.1 to 13.2 g/capita/day), and by 2 g/capita/day between the Phase 1 and Phase 2 periods (from 13.5 to 10.4 g/capita/day), totaling a 5.9 g/capita/day overall decrease. Between the pre-policy period and Phase 1, reformulation accounted for 28% (− 1.10 g/capita/day) of the reduction in sugar purchases, while changes in purchase volume accounted for 30% (− 1.19 g/capita/day). In contrast, from Phase 1 to Phase 2, the reduction was largely driven by consumer responses, with switching accounting for 55% (− 1.10 g/capita/day) and changes in purchase volume accounting for 42% (− 0.80 g/capita/day). Across all phases, producer responses, namely reformulation (19%) and net product entry (7%), were smaller than consumer responses, namely purchase volumes (36%) and switching between products (11%). The ‘other’ component captures the cross terms and residuals from decomposition equations. The overall negative value indicates that lower-sugar beverages gained market share or higher-sugar beverages lost market share.

The majority of the results above are driven by shifts in the beverages high in sugar (Appendix A4). Specifically, the sugar content of high in sugar beverage purchases decreased by 3.5 g/capita/day from the Pre-policy to Phase 1 period (from 13.0 to 9.5 g/capita/day), and by 1.1 g/capita/day between Phase 1 and Phase 2 (from 9.5 to 8.-4 g/capita/day), resulting in a total decrease of 4.6 g/capita/day. Similar to the overall results, after some initial reformulation, consumer responses were primarily responsible for reductions in sugar content among high-sugar beverages in both phases. For the low sugar beverages, the sugar content of beverage purchases fell by less than 0.2 g/capita/day across all policy periods, relative to an average of 5 g/capita/day in any given period. Despite these small net changes, volume effects accounted for the largest share of the reduction, followed by product switching. As expected, we observe minimal reformulation among products not directly targeted by the policy.

### Secondary analysis

When disaggregating the changes between higher and lower SES households, we observe similar results and underlying factors as shown in Fig. 4. Specifically, in the higher SES households the sugar content of beverage purchases decreased by 4.9 g/capita/day from the Pre-policy to Phase 1 period (from 17.6 to 12.7 g/capita/day), and by 2.5 g/capita/day between Phase 1 and Phase 2 (from 12.7 to 10.1 g/capita/day), resulting in a total decrease of 7.5 g/capita/day (from 17.6 to 10.1 g/capita/day). Similarly in the lower SES households the sugar content of beverage purchases decreased by 3.6 g/capita/day from the pre-policy to Phase 1 period (from 17. to 13.3g/capita/day), and by 1.8 g/capita/day between Phase 1 and Phase 2 (from 13.3 to 11.4 g/capita/day), resulting in a total decrease of 5.5 g/capita/day (from 17 to 11.4 g/capita/day). For both high- and low-SES groups, cumulative reductions were largely driven by changes in purchase volumes. Reformulation contributed most between the pre-policy and Phase 1 periods, whereas product switching was more influential between Phase 1 and Phase 2.

## Discussion

This study decomposes changes in the sugar content of beverage purchases into producer-and consumer-driven components. On the producer side, we observe a decline in the introduction of new beverages and substantial beverage discontinuation following Phase 1 implementation of Chile’s Front-of-Package Label policy. Although the percentage of unique beverage products that were reformulated were limited, once we account for purchase volumes, the resulting overall reductions in sugar are substantial. Reformulation contributed most during Phase 1, while reductions in purchase volume and increased product switching played a larger role in Phase 2. Together, these findings suggest that changes in both producer behavior and consumer purchasing contributed to reductions in population-level sugar exposure following policy implementation. We discuss each of these findings below.

New beverage introductions declined across policy phases, with a larger reduction among high-sugar than low-sugar beverages, likely reflecting greater caution around products subject to the regulation or anticipated reductions in consumer demand. Substantial beverage discontinuation was observed across policy phases, including among low-sugar beverages. This may partly reflect the inherently dynamic nature of the beverage market, where high SKU turnover is common as brands experiment with formats, flavors, and packaging. This is consistent with broader market dynamics, where Euromonitor International report that nearly one-third of fast-moving consumer goods brands, of which beverages are a major subset, launched in 2022 were discontinued by the end of 2023 [21]. Some discontinued products may also have been reformulated and reintroduced under new SKUs, contributing to the observed discontinuation rates even when the underlying product type remained available. Such product discontinuation and relaunch may represent an alternative to reformulation. Firms may discontinue products rather than modify their nutritional composition, while introducing new SKUs with improved nutritional profiles but similar positioning. The relatively high number of new product introductions in Phase 1, particularly among low-sugar beverages, is consistent with this possibility, although our data cannot distinguish relaunches from genuinely new products.

Most continuing beverage products were not reformulated, with the majority retaining their original sugar levels through Phase 2. Among reformulated products, sugar reductions were more common than increases, although some high-sugar beverages increased their sugar content, particularly during Phase 1. The tiered structure of Chile’s sugar-sweetened beverage tax may provide one possible context for this pattern, as products already subject to the highest tax rate could increase their sugar content without incurring a higher tax rate as long as they remained within the same tax tier. However, our data do not allow us to determine whether tax incentives contributed to these changes. Additionally, while policy efforts often focus on supply-side reformulation, consumer preferences can limit their impact. If reduced-sugar versions were perceived as less palatable, companies may have decided to revert to original or higher-sugar formulations to maintain market share. Although we cannot directly test pre-policy reformulation, existing evidence suggests that industry response in anticipation of policy was limited. Fewer than 2% of products in major Santiago supermarkets were reformulated before implementation specifically to avoid warning labels, despite projections that most packaged products would exceed at least one nutrient threshold [22].

Consistent with our findings, Reyes et al. [7] found that following Chile’s warning-label law, the share of food and beverage products exceeding the sugar threshold declined from 80% to 60%, with nutrient levels clustering just below regulatory cutoffs, suggesting reformulation in phase 1 to avoid warning labels. This clustering suggests that discrete thresholds may encourage firms to reduce sugar just enough to avoid a warning label, highlighting the potential value of more continuous thresholds or graduated incentives for further reductions. Reformulation was often accompanied by increased use of non-nutritive sweeteners, suggesting that manufacturers-maintained sweetness while meeting labeling thresholds [23, 24].

Evidence from other Latin American countries similarly suggests that FOP labeling policies can prompt product reformulation. In Peru [25] and Mexico [14], mandatory warning labels on pre-packaged foods and non-alcoholic beverages were associated with product reformulation, particularly reductions in sugar and calorie content and a smaller share of products exceeding labeling thresholds. In Ecuador, where traffic-light labels indicate high, medium, or low levels of sugar, fat, and salt, sugar content in soft drinks declined by approximately 13% following implementation [26].

Our results show, that in Chile, most reformulation was driven largely by high-sugar beverages, while minimal reformulation was observed among products not directly targeted by the policy. This aligns with evidence from South Africa, where reformulation and sugar reductions were concentrated among taxable beverages [17, 27], as well as evidence from the UK Soft Drinks Industry Levy, where the proportion of soft drinks exceeding the 5 g/100 mL levy threshold declined substantially following implementation [28].

However, product-level changes do not necessarily translate directly into changes in sugar purchases, as their contribution depends on how much consumers purchase of each product. Our decomposition, which accounts for purchase volume, suggests that in Phase 1, sugar reductions reflected both consumer and producer responses, with notable effects from changes in purchase volumes alongside reformulation. By Phase 2, however, reductions were driven primarily by consumer behavior, mainly lower purchase volumes and product switching. Volume changes accounted for a substantial portion of the sugar reduction, with 30% occurring between the pre-policy period and Phase 1, and much of this persisted over time. By Phase 2, consumers’ product switching became increasingly important, explaining 56% of the reduction compared with 42% attributable to volume changes over the same period. These patterns indicate not only sustained consumer behavior change but also a strengthening of such responses over time in the second phase.

These shifts likely reflect the gradual influence of broader social and regulatory changes in Chile. As concurrent labeling, marketing restrictions, and school-based regulations in Chile reshaped norms and expectations around sugary beverages, consumer preferences may have progressively adapted rather than plateauing due to labelling fatigue [29]. The persistence of these effects may also reflect Chile’s consistent, phased implementation of complementary policies, although our study cannot isolate the contribution of individual policy instruments. This suggests that sustained and integrated policy implementation may be important for supporting longer-term changes in consumer behavior. Evidence from other countries supports this interpretation. In Mexico, larger reductions in SSB purchases were observed in the second year of the tax relative to the first, indicating a deepening consumer response over time [30].

Although no clear patterns emerged by socioeconomic status, higher-SES households experienced a somewhat larger overall absolute reduction in sugar content (7.5 g/capita/day) compared to lower-SES households (5.5 g/capita/day), with both groups showing similar decomposition patterns. These patterns carry important public health implications, suggesting that mandatory labeling policies can shift purchasing behaviors in both higher- and lower-SES households and may therefore contribute to population-wide reductions in sugar intake, even if absolute changes vary. A key limitation of this analysis is that not all continuing beverage items were purchased by both socioeconomic groups in each period, and likewise, new or discontinued items were not consistently purchased by both groups during the periods when they were introduced or removed.

## Conclusions

Chile’s implementation of front-of-package labeling was associated with a substantial reduction in the sugar content of beverage purchases. Our findings indicate that most of the overall reductions occurred after Phase 1 and were largely driven by changes in high sugar beverages. In Phase 1, both consumer and producer responses contributed relatively equally to the overall reduction. In contrast, in Phase 2, consumer behavior played a larger role, with product switching and reduced purchase volumes driving most of the reductions. Reformulation contributed only a small share, suggesting that most reformulation-led reductions were limited to initial phase of policy implementation. While no clear patterns emerged across socioeconomic groups, high socioeconomic households experienced a slightly greater total reduction compared to low socioeconomic households, though both showed similar decomposition patterns. Consistent with existing evidence, we find that nutrition policies can create incentives for producers to reformulate and reduce nutrients of concern. At the same time, our findings highlight the important role of consumer responses, particularly changes in product choice and purchase volume, in shaping the overall impact of the policy. These findings underscore the importance of considering both supply- and demand-side mechanisms when designing effective food policies.

## Supplementary Material

This is a list of supplementary files associated with this preprint. Click to download.

• AdditionalFile1Appendix.docx

## Figures and Tables

**Figure 1:**
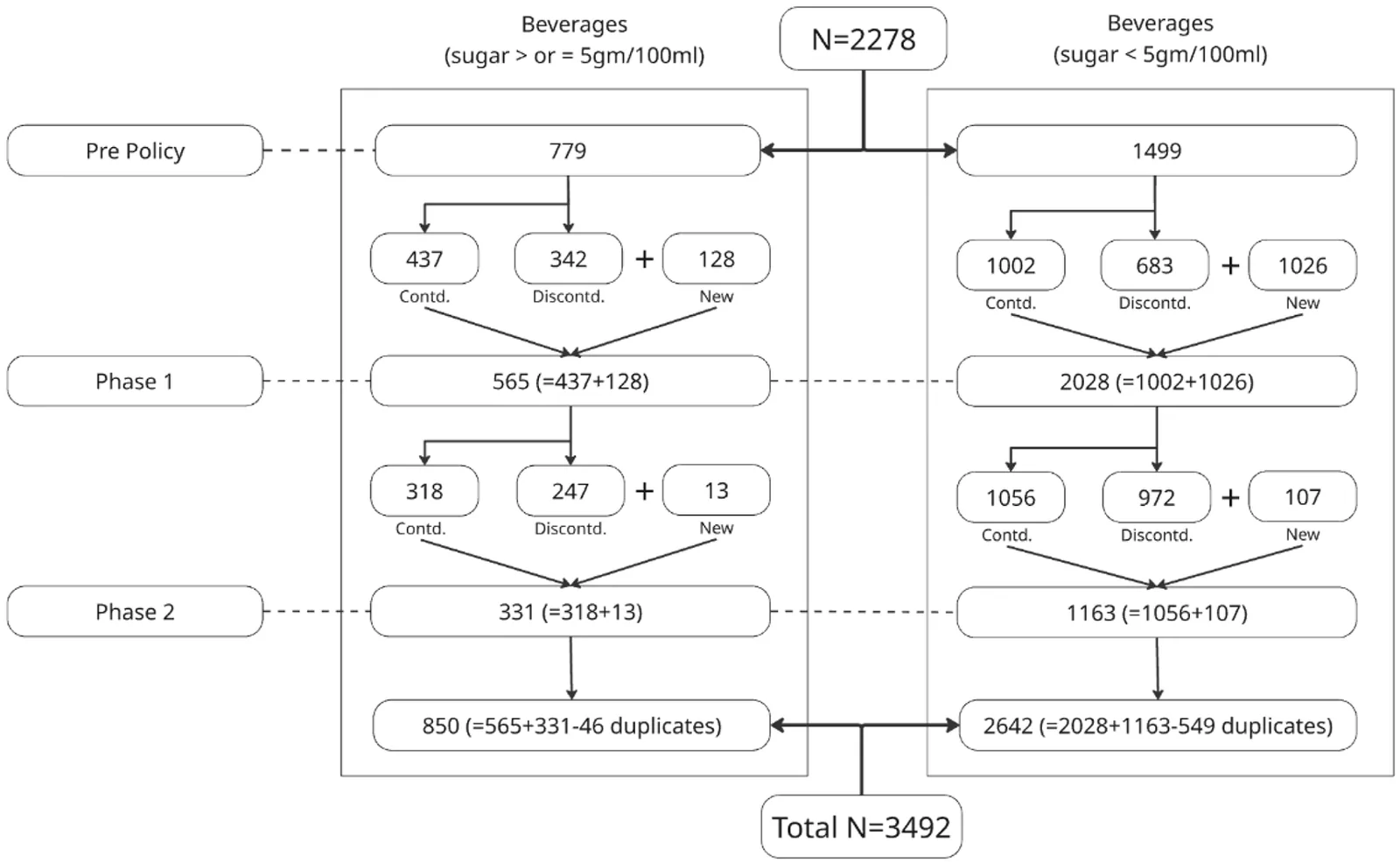
Continuing, New, and Discontinued Beverage Items across Policy Phases **Note:** 1. The pre-policy period ended in June 2016, followed by Phase 1 (July 2016 to June 2018) and Phase 2 (July 2018 onward) 2. Continuing items are items that were purchased in the current and last periods; new items are items that were purchased in the current period but not last period; discontinued items are items that were purchased last period but not in the current period and are therefore excluded from a period’s totals. 3. Duplicates refer to products that were available throughout the period and are therefore counted in Pre-policy and Phase 1 and are hence subtracted from the total product count. 4. For high-sugar beverages, 437 products continued into Phase 1 (432 with complete information) and 318 into Phase 2 (271 complete). For low-sugar beverages, 1,002 continued into Phase 1 (972 complete) and 1,056 into Phase 2 (1,053 complete). All products were retained to capture turnover, but only those with complete information were included in reformulation and decomposition analyses. Source: Authors’ calculations based on Chile’s household purchase data from Kantar Panel Data (Jan 2015-June 2019).

**Figure 2:**
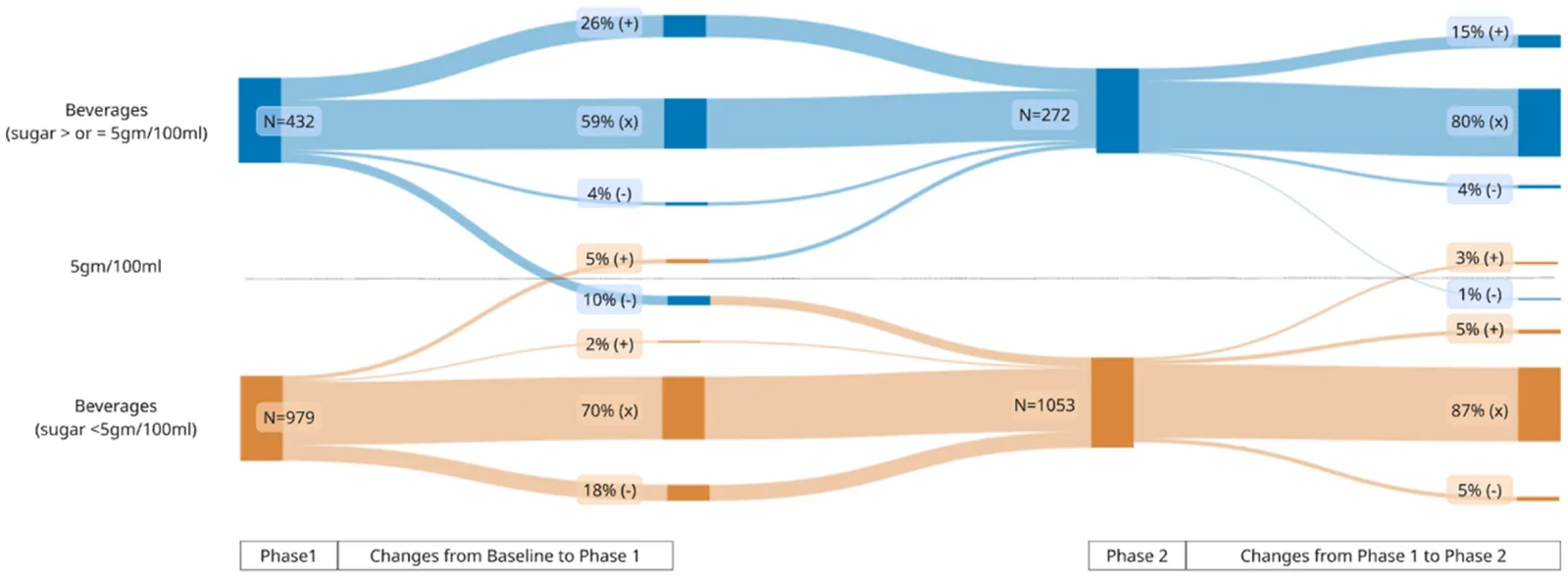
Reformulation in Continued Products **Note:** 1. The pre-policy period lasted until June 2016, followed by Phase 1 from July 2016 to June 2018, and Phase 2 beginning in July 2018 onwards. Reformulation defined as changes in sugar concentration (rounded to the nearest 0.1 g/100 ml) greater than 20%. Source: Authors’ calculations based on Chile’s household purchase data from Kantar Panel Data (Jan 2013-June 2019). 2. For high-sugar beverages, 437 products continued into Phase 1 (432 with complete information) and 318 into Phase 2 (271 complete). For low-sugar beverages, 1,002 continued into Phase 1 (972 complete) and 1,056 into Phase 2 (1,053 complete). All products were retained to capture turnover, but only those with complete information were included in reformulation and decomposition analyses.

**Figure 3:**
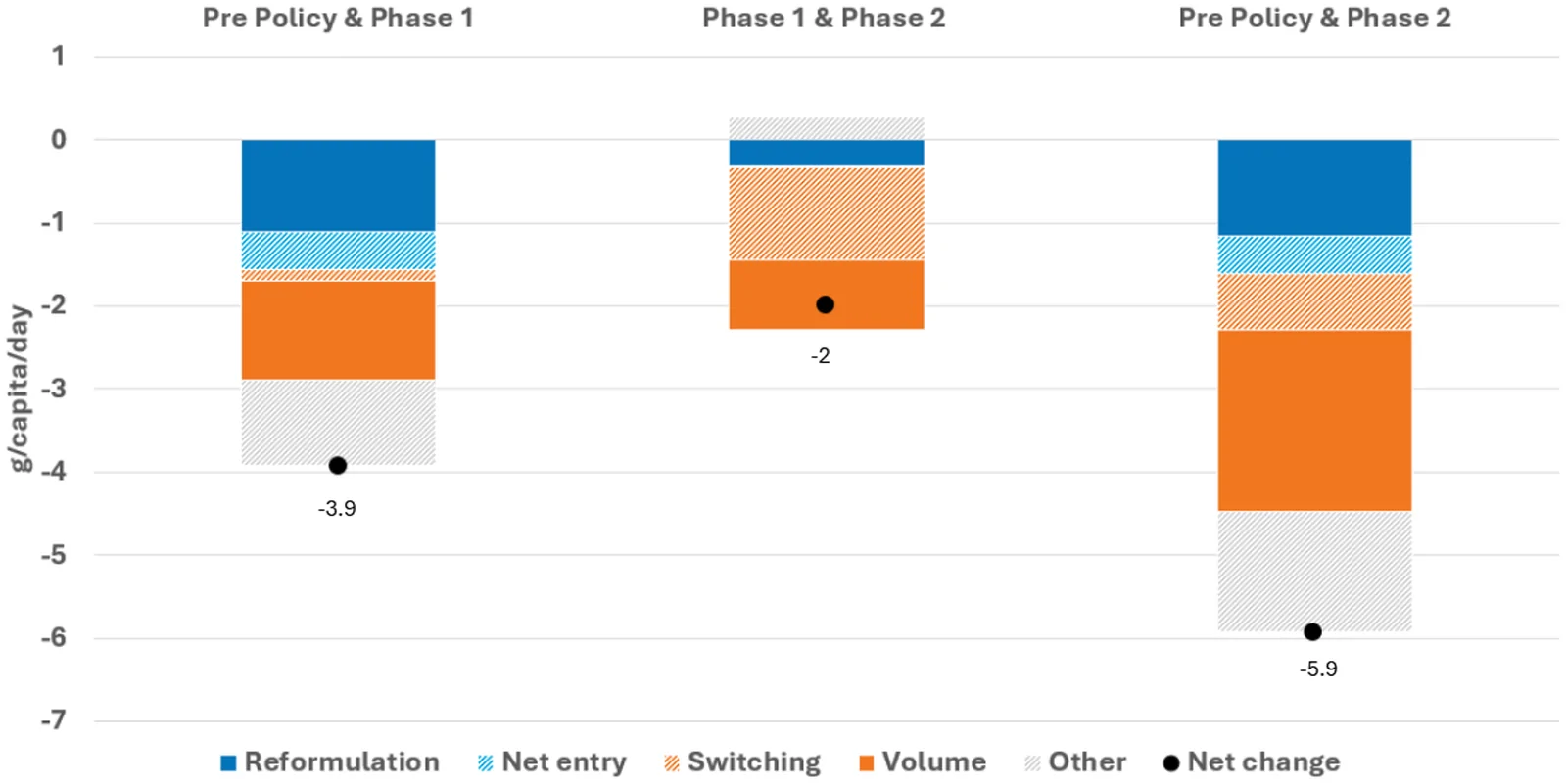
Decomposition of Changes in the Sugar Content of Non-Alcoholic Beverage Purchases (g/capita/ day) Note: 'Volume' represents the part of the net change accounted for by changes in the volume of beverage purchases; 'reformulation' represents the part of the net change accounted for by the reformulation of continuing beverages by producers; 'switching' represents the part of the net change accounted for by switching between beverages by consumers (within and across taxable categories); 'net entry' represents the part of the net change accounted for by the introduction of new beverages and the discontinuation of existing beverages by producers; 'cross terms' captures the cross terms in both decomposition stages correcting for simultaneous volume and sugar concentration changes.

**Figure 4:**
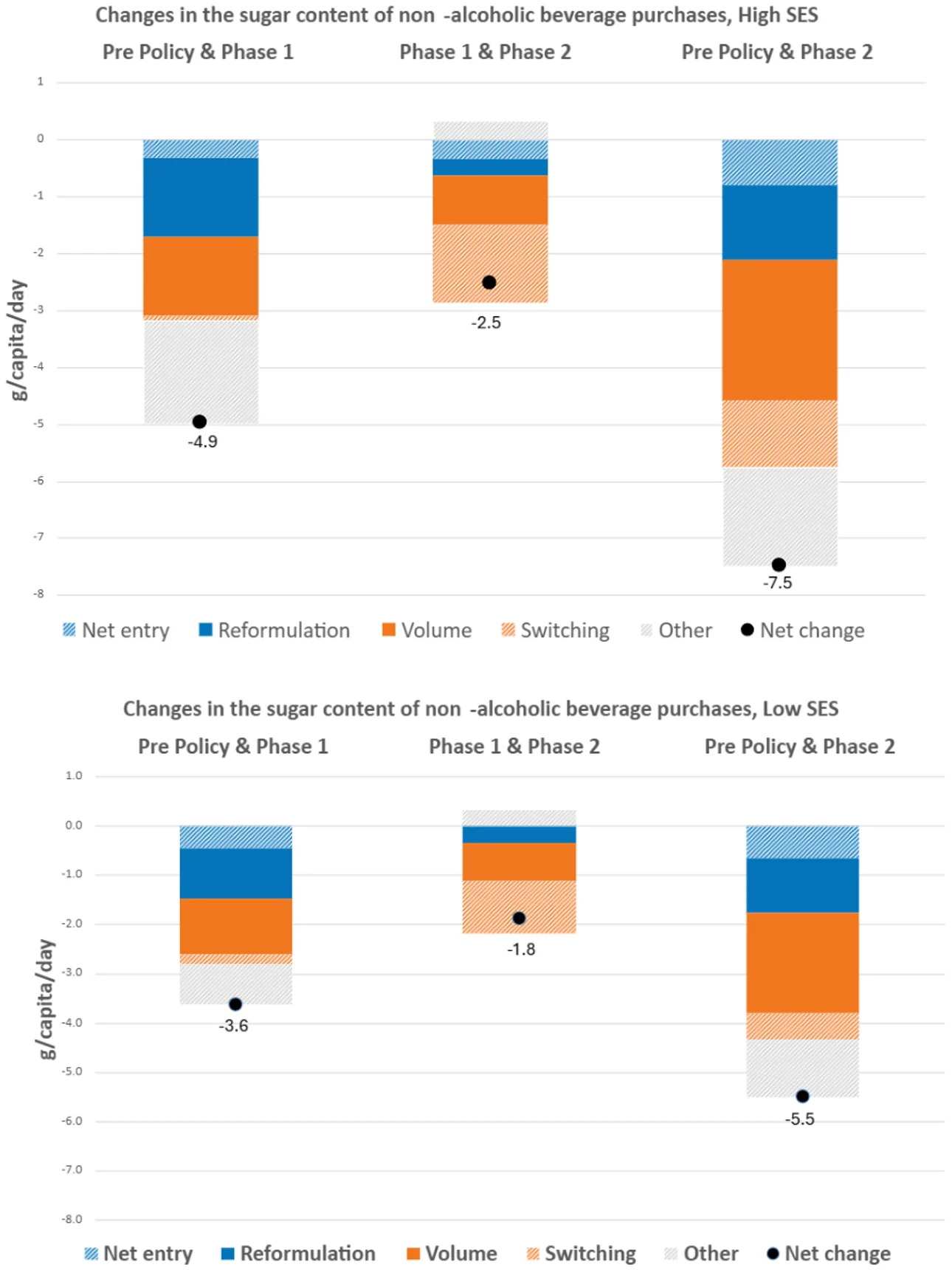
Decomposition of Changes in the Sugar Content of Non-Alcoholic Beverage Purchases (g/capita/ day)-By SES
